# Home Spirometry for Post-COVID Recovery: A Clinical Validation Study of an Ultrasonic Device

**DOI:** 10.3390/diagnostics15111396

**Published:** 2025-05-30

**Authors:** Asli Gorek Dilektasli, Ayten Odabas, Ismet Polat, Abdurrahman Dogan, Guven Ozkaya, Ozge Aydin Guclu, Nilufer Aylin Acet Ozturk, Funda Coskun, Mehmet Karadag

**Affiliations:** 1 Department of Pulmonary Medicine, Faculty of Medicine, Bursa Uludag University, 16059 Bursa, Turkey; aytenodabas@uludag.edu.tr (A.O.); ismetpolat@uludag.edu.tr (I.P.); abdurrahmandogan@uludag.edu.tr (A.D.); ozgeguclu@uludag.edu.tr (O.A.G.); nacet@uludag.edu.tr (N.A.A.O.); fundacoskun@uludag.edu.tr (F.C.); karadag@uludag.edu.tr (M.K.); 2 Pulmonary Rehabilitation Unit, Department of Pulmonary Medicine, Faculty of Medicine, Bursa Uludag University, 16059 Bursa, Turkey; 3 Department of Biostatistics, Faculty of Medicine, Bursa Uludag University, 16059 Bursa, Turkey; guvenozkaya@gmail.com

**Keywords:** home spirometry, post-COVID pulmonary functions, telemonitoring, eHealth

## Abstract

**Background/Objectives:** Patients recovering from COVID-19 often experience persistent respiratory symptoms, necessitating pulmonary function monitoring. While clinical spirometry is the gold standard, home spirometry offers a remote alternative. This study evaluated the validity of an ultrasonic home-based spirometer for monitoring lung function in post-COVID-19 pneumonia patients over 12 weeks. **Methods**: This prospective study included 30 post-COVID pneumonia patients who underwent clinical spirometry at weeks 4, 8 and 12. Participants performed weekly home spirometry using the SpiroHome Personal^®^ device. Agreement between home and clinical spirometry was assessed using a Bland–Altman analysis, intraclass correlation coefficients (ICCs), and Pearson correlation coefficients. Pulmonary function changes over time were analyzed using repeated measures ANOVA. **Results**: Home spirometry showed strong agreement with clinical spirometry for forced vital capacity (FVC) and forced expiratory volume in the first second (FEV1), with ICC values exceeding 0.92. The Bland–Altman analysis demonstrated minimal bias, though limits of agreement exceeded the clinically accepted threshold of ±150 mL. FEV1/FVC ratios showed greater variability. Pulmonary function improved significantly over 12 weeks for both methods (*p* < 0.002). Patient adherence to home spirometry remained high, with a median of 18.50 sessions [IQR: 15.00–26.00] and an overall compliance rate of 98.33% ± 9.13%. **Conclusions**: Home spirometry provides reliable pulmonary function measurements, particularly for FVC and FEV1, supporting its role as a remote monitoring tool. Despite minor variability in FEV1/FVC, home spirometry enables frequent assessment of lung function recovery, potentially reducing hospital visits and improving patient management.

## 1. Introduction

Patients recovering from acute COVID-19 often experience a wide range of lingering symptoms, collectively referred to as “long COVID” or “post-COVID conditions” [1,2,3,4]. These symptoms may include persistent dyspnea, fatigue, chest discomfort, and exercise intolerance, all of which can significantly impact daily life and overall quality of health [3,5]. Post-COVID pulmonary consequences are particularly alarming, as COVID-19 predominantly impacts the lungs, frequently resulting in pneumonia, acute respiratory distress syndrome, and long-term lung function deterioration [3,4,6].

Pulmonary function testing (PFT) is essential for evaluating lung recovery in patients with persistent respiratory symptoms following their initial infection. International guidelines recommend that individuals recovering from COVID-19 with persistent, progressive, or new-onset respiratory symptoms should undergo pulmonary function testing to evaluate lung function and detect potential sequelae such as restrictive or obstructive impairments [7,8,9]. However, the optimal timing for performing PFTs in post-COVID patients remains uncertain [10,11]. Based on the existing literature and experience with acute respiratory distress syndrome unrelated to COVID-19, PFTs are generally recommended between 6 to 12 weeks post-hospital discharge [10,11,12,13]. Despite improvements in symptoms over time, studies suggest that a significant proportion of recovered COVID-19 patients exhibit persistent lung function abnormalities, particularly those with severe pulmonary involvement during their acute illness [14]. Numerous investigations evaluating post-COVID pulmonary function have documented the impaired diffusing capacity of the lungs for carbon monoxide (DLCO), restrictive ventilatory abnormalities, and diminished forced vital capacity (FVC) [7,14,15,16]. Given the significant burden of post-COVID lung sequelae, pulmonary function monitoring is essential for identifying patients at risk of long-term impairment and guiding rehabilitation strategies [9,15]. Traditional PFTs require patients to visit specialized pulmonary function laboratories, which may pose logistical challenges and increase healthcare system burdens [11,13]. The COVID-19 pandemic has further emphasized the need for remote monitoring solutions, including home-based spirometry, to facilitate continuous pulmonary function assessment without necessitating frequent hospital visits [17].

Home spirometry has emerged as a promising alternative for evaluating lung function outside clinical settings, enabling frequent and convenient monitoring of respiratory parameters [18]. Portable home spirometry devices allow patients to track forced expiratory volume in the first second (FEV_1_), forced vital capacity (FVC), and FEV_1_/FVC ratios, offering critical insights into pulmonary recovery and disease progression [19]. Recent studies have demonstrated that home spirometry correlates well with standard laboratory-based spirometry, offering high reliability and ease of use for patients with chronic respiratory diseases such as asthma and chronic obstructive pulmonary disease (COPD) [20,21]. However, data on the validity and clinical utility of home spirometry in post-COVID patients remain limited.

Sekerel et al. validated the SpiroHome Clinical ultrasonic spirometer, confirming its adherence to ATS/ERS standards for accuracy and repeatability in clinical settings [22]. Additionally, Ilic used the home spirometry device for home monitoring in interstitial lung disease patients, and Bell validated its use for adults with cystic fibrosis [23,24]. Both studies reported that the device produced acceptable and repeatable results. However, the clinical validation of the home-based version of SpiroHome for post-COVID pulmonary function monitoring has not been thoroughly investigated.

This study aimed to evaluate the clinical validity of an ultrasonic home-based spirometer for monitoring pulmonary function in post-COVID patients over 12 weeks. We hypothesized that home spirometry would provide reliable and reproducible lung function measurements comparable to clinical spirometry, thereby offering an effective alternative for remote respiratory monitoring in this patient population. Evaluating the feasibility and accuracy of home-based spirometry in COVID-19 survivors could help facilitate early detection of lung function deterioration and guide personalized rehabilitation interventions.

## 2. Materials and Methods

### 2.1. Study Design and Population

This prospective study included post-COVID pneumonia patients (PCR-confirmed) referred for spirometry at Bursa Uludag University, Department of Pulmonary Medicine, between April and December 2021. This study was designed as a prospective feasibility study, and a formal sample size calculation was not performed. Instead, the number of participants was determined based on practical considerations, including patient availability during the study period and resource limitations, which are typical of pilot or exploratory studies in this context. The study protocol was approved by the Institutional Review Board and Ethics Committee (2011-KAEK-26/509), and informed consent was obtained from all participants. Patients underwent clinic-based spirometry at baseline and weeks 4, 8, and 12. At the baseline visit, participants received a home spirometer with comprehensive training and were instructed to perform weekly home spirometry for 12 weeks.

### 2.2. Clinical Spirometry

Patients performed clinical spirometry with the VyntusTM One (Vyntus Spiro, Carefusion, Yorba Linda, CA, USA) device. Calibration procedures: The Vyntus device underwent daily calibration, and volume calibration procedures were conducted daily using a certified 3-L syringe, following ATS/ERS guidelines [25]. All of the participants received standardized instructions while performing spirometry. All spirometry measurements followed the 2019 ATS/ERS guidelines [25]. The same skilled technician conducted the spirometry tests. Participants were instructed to execute 3– 8 forced expiratory maneuvers to fulfill the acceptability and reproducibility criteria. At each clinic visit, a trained respiratory technician evaluated the participant’s performance and provided re-training if the quality grading of the spirograms dropped below B or if reproducibility criteria were not met. Each visit lasted approximately 30–45 min to ensure time for instruction, rest between maneuvers, and an explanation of the results.

### 2.3. Home Spirometry

Participants used the SpiroHome Personal^®^ (Inofab Health, Ankara, Turkey), a CE-certified, portable ultrasonic spirometer that pairs with a smartphone or tablet via Bluetooth^®^. Spirometry data were uploaded to the SpiroCloud^®^ platform in real-time, where study investigators could review the results. The cloud platform provides a full spirometry report with all of the maneuvers performed by the patient, as well as flow-volume and volume-time graphs for each. The device’s software determined the best maneuver and quality grades for FVC and FEV_1_, following the recommendations of ATS/ERS [25]. At the baseline visit, each participant received in-person training from a study investigator, which included: (1) a live demonstration of the home spirometer; (2) viewing of a short instructional video; (3) guided practice with real-time feedback; and (4) confirmation of understanding through successful performance of three acceptable and repeatable FVC maneuvers (variation ≤ 150 mL). A patient was considered successfully trained if they produced three acceptable and repeatable FVC measurements that were acceptable and repeatable, with the highest FVC values differing ≤150 mL. This hands-on session lasted approximately 45 min per participant. A researcher created the SpiroCloud^®^ account on each participant’s mobile device and ensured proper device pairing.

Participants were instructed to perform home spirometry once per week, on a predetermined day (3–8 maneuvers per session) between 09:00–12:00 throughout the 12-week study period. A research nurse monitored weekly adherence via the cloud platform. The application provided guidance, instructions, and feedback to perform acceptable and repeatable spirometry maneuvers. During each visit, participants were re-trained to perform proper home spirometry if their performance fell below the acceptable quality control standards (below A) and if it was determined that their compliance with home spirometers was low. Adherence was defined as completing ≥3 weekly maneuvers for 12 weeks. All training sessions and performance reviews followed ATS/ERS guidelines. Study personnel maintained logs of training outcomes and retraining interventions to ensure consistent application of quality standards.

### 2.4. Quality Control and Spirometry Parameters

Forced vital capacity (FVC), forced expiratory volume in the first second (FEV_1_), and FEV_1_/FVC, along with their respective z-scores according to the Global Lung Initiative (GLI) reference equations were recorded [26]. The highest values for FVC and FEV_1_ across all accepted maneuvers were included in the final analysis. Spirometry quality grading (ranging from A to F) was assigned based on ATS/ERS criteria by both the Vyntus™ One and SpiroHome software. Grades were defined as follows: Grade A: ≥3 acceptable measurements within 0.150 L, with the two highest FVC values within 150 mL; Grade B: 2 acceptable measurements within 0.150 L; Grade C: ≥2 acceptable measurements within 0.200 L; Grade D: ≥2 acceptable measurements within 0.250 L, Grade E: ≥2 acceptable measurements with a difference > 0.250 L; and Grade F: no acceptable or usable maneuvers. FVC and FEV_1_ measurements were included in the final analysis, regardless of their quality rating, to ensure maximum generalizability.

### 2.5. Patient Satisfaction

At week 12, participants completed an online survey (developed according to the European Lung Foundation recommendations) to assess their experience with home and clinical spirometry [27]. The survey included questions about spirometry test experience (regarding information and recommendations received), spirometry preferences (mouthpieces, nose clips, and test duration), as well as spirometry-related discomfort and adverse effects related to both home spirometry and clinical spirometry testing.

### 2.6. Statistical Analysis

The characteristics of the participants were reported using mean values and standard deviations (SD), or median values and the interquartile range [IQR 25–75%] for continuous variables. Categorical data were presented as percentages. Statistical analysis was performed by using IBM SPSS Statistics for Windows, Version 29.02.0, Armonk, NY, USA: IBM Corp, and MedCalc for Windows, Version 23.0.2 (MedCalc Software, Ostend, Belgium).

Agreement between home spirometry (HS) and clinical spirometry (CS) was assessed using a Bland–Altman analysis [28], which evaluates bias (mean difference) and limits of agreement (LoA). The mean difference (bias) was calculated as CS—HS, and the 95% LoA were determined as follows: Mean Difference ± 1.96 × SD of Differences. Bland–Altman plots were generated to visualize systematic bias and variability across different measurement ranges. A narrow LoA indicates strong agreement between the two methods.

The intraclass correlation coefficient (ICC) was calculated to assess the reliability and consistency between home and clinical spirometry measurements. A two-way random-effects model (absolute agreement type ICC) was used. The ICC values were interpreted as follows: ICC ≥ 0.90: excellent agreement, ICC 0.75–0.89: good agreement, ICC 0.50–0.74: moderate agreement, and ICC < 0.50: poor agreement. The 95% confidence intervals (CI) for ICC were computed using standard error estimation. A high ICC with a narrow CI suggests strong agreement and low variability between the two measurement methods. Pearson correlation coefficients were calculated to examine the linear relationship between clinical and home spirometry measurements. However, correlation does not assess bias or agreement, so it was reported alongside the Bland–Altman and ICC analyses for reference.

Validity was evaluated by comparing home and clinical spirometry values. Absolute differences in FVC and FEV_1_ were analyzed using the paired *t*-test (normally distributed data).

Changes in pulmonary function over time were analyzed using repeated measures ANOVA, with Greenhouse–Geisser correction when the sphericity assumption was violated. The McNemar test was used to compare patient feedback on home spirometry versus clinical spirometry, assessing differences in user experience and preferences between the two methods. An overall α = 0.05 type-I error level was used to infer statistical significance.

## 3. Results

### 3.1. Study Population

Initially, 34 post-COVID pneumonia patients were invited to participate; however, two patients withdrew during the first week, and two others passed away during follow-up. The final study cohort comprised 30 patients, with a mean age of 48.64 ± 10.49 years, of whom 30% were female. Among them, 96.7% (*n* = 29) had PCR-confirmed SARS-CoV-2 infection, 80% (*n* = 24) had bilateral lung involvement, and 76.7% (*n* = 23) required hospitalization during acute COVID-19. At baseline, the mean forced vital capacity (FVC) was 3.20 ± 0.99 L, with a z-score of −1.62 ± 1.86, and a predicted FVC% of 83.93 ± 22.11% (Table 1). At total of 31.0%, 23.3%, and 46.7% of the study participants completed primary school, high school, and university, respectively. The quality grading for FVC and FEV_1_ by both clinical and home spirometry per visit is summarized in Appendix A.1. The quality of FVC and FEV_1_ measurements was assessed for both clinical and home spirometry at weeks 4, 8 and 12. For clinical spirometry, Grade A test quality was achieved in 53.8% to 82.8% of FVC tests and 59.0% to 79.3% of FEV_1_ tests. For in-home spirometry, Grade A quality was observed in 63.3% to 76.7% of FVC tests and 63.3% to 83.3% of FEV_1_ tests. When considering at least Grade B quality, clinical spirometry achieved 69.0% to 96.6% for FVC and 74.4% to 96.5% for FEV1. Similarly, home spirometry demonstrated 83.3% to 96.7% for FVC and 70.0% to 90.0% for FEV_1_.

### 3.2. Compliance with Home Spirometry

Throughout the 12 weeks, each participant recorded a median of 18.50 home spirometry sessions [IQR: 15.00–26.00]. The number of recorded measurement days decreased over time: Week 0–4: 7.00 [6.00–10.25], Week 4–8: 5.00 [4.00–7.00], and Week 8–12: 5.00 [4.00–7.00], (*p* < 0.0001). The number of measurement days in the first four weeks was higher than those from weeks 4 to 8 and weeks 8 to 12 (*p* = 0.005 and *p* < 0.0001). Despite the decline, patient adherence remained high, averaging 98.33% ± 9.13% across all study periods.

### 3.3. Agreement Between Clinical and Home Spirometry

**Figure 1:**
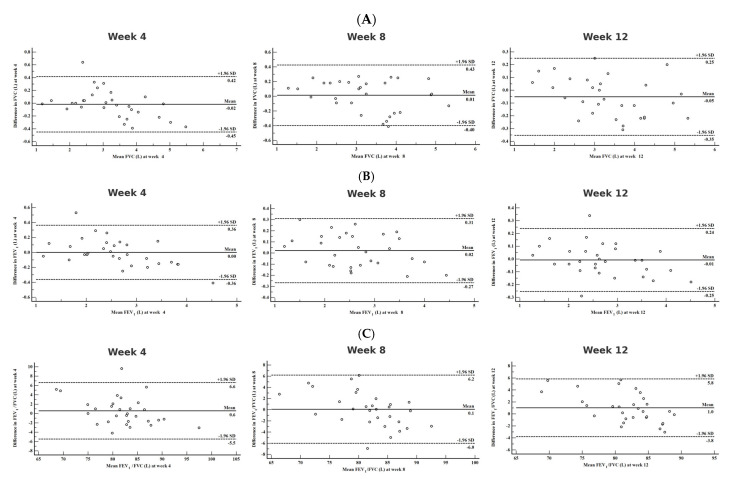
Bland-Altman Plots for the agreement between home and clinical spirometry. Panels (**A**–**C**) present Bland-Altman plots illustrating the agreement between home spirometry and clinical spirometry measurements for forced vital capacity (FVC), forced expiratory volume in the first second (FEV_1_), and the FEV_1_/FVC ratio at weeks 4, 8, and 12. Each plot depicts the mean difference (bias) between the two methods along with the 95% limits of agreement (dashed lines). Panel (**A**) (FVC): The three Bland-Altman plots demonstrate the agreement between FVC values obtained from home and clinical spirometry at weeks 4, 8, and 12. The mean bias is close to zero, and most data points fall within the 95% limits of agreement, suggesting good agreement across all time points. Panel (**B**) (FEV_1_): Similar to FVC, the plots show strong agreement between home and clinical spirometry for FEV_1_ across the three time points. The limits of agreement remain relatively consistent over time, with minimal bias observed. Panel (**C**) (FEV_1_/FVC Ratio): The agreement between home and clinical spirometry for the FEV_1_/FVC ratio is also presented at weeks 4, 8, and 12. The plots show that most values fall within the 95% limits of agreement, although some variability is present.

The Bland–Altman plots comparing clinical spirometry-based and home spirometry-based readings demonstrate a good level of agreement (Figure 1A–C), with no significant systematic bias observed. The limit of agreement values exceed acceptable clinical limits (>±150 mL for FVC and FEV_1_).

The mean bias (mean difference) across all time points is small, suggesting minimal systematic differences between the two devices (Table 2). Clinical spirometry measurements for FVC at 4 weeks (−0.02 L) and 12 weeks (−0.05 L), as well as FEV_1_ at 12 weeks (−0.01 L), tend to be slightly lower than home spirometry. These minor differences can likely be attributed to within-maneuver variation, which remains within clinically acceptable limits.

Intraclass correlation coefficients confirm a high level of reliability and agreement between clinical and home spirometry, with ICC values exceeding 0.92 for all comparisons (Table 2). The 95% confidence intervals for ICC remain above 0.97 for FVC and FEV_1_, while FEV_1_/FVC shows slightly more variability, with ICC values above 0.86, still indicating strong consistency.

For z-scores of spirometric parameters, mean differences across all comparisons remain small, further confirming no significant systematic bias between clinical and home spirometry (Table 3). However, Z-FVC at 12 weeks exhibited a slightly larger bias compared to other time points, suggesting minor deviations in measurement consistency. FEV_1_/FVC measurements showed more variability in limits of agreement, potentially indicating that home spirometry may be slightly less precise in assessing airflow limitation.

ICC values for z-scores of FVC were consistently ≥ 0.87, indicating good agreement between clinical and home spirometry (Table 3). However, z-score ICC values for FEV_1_ ranged from 0.45 to 0.86, and FEV_1_/FVC ranged from 0.47 to 0.83, indicating poor to good agreement depending on the time point. The strongest agreement was observed in Z-FVC and Z-FEV_1_ comparisons, reinforcing the accuracy of home spirometry for tracking lung volume recovery.

### 3.4. Correlations Between Clinic and Home Spirometry Measurements

**Figure 2:**
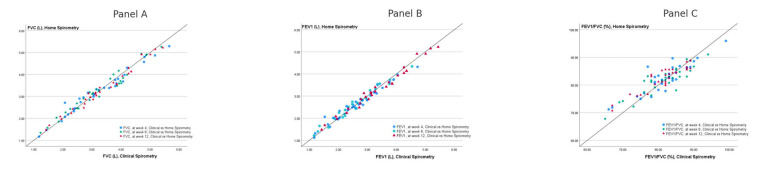
Correlation between clinical and home spirometry measurements at weeks 4, 8, and 12. Scatter plots comparing spirometric measurements obtained from clinical and home spirometry at weeks 4, 8, and 12. Panel (**A**) shows forced vital capacity (FVC), Panel (**B**) forced expiratory volume in the first second (FEV_1_), and Panel (**C**) represents the FEV_1_/FVC ratio. Each data point corresponds to an individual measurement, with colors and shapes distinguishing different time points: blue circles for week 4, green stars for week 8, and red triangles for week 12. The diagonal line represents the line of identity (y = x), indicating perfect agreement between clinical and home spirometry measurements. The close alignment of data points along this line suggests strong agreement between the two measurement methods across all time points. In Panels (**A**,**B**), the data points are closely aligned along the identity line (y = x), indicating a strong correlation between the FVC and FEV1 methods. The scatterplot in Panel (**C**), evaluating FEV1/FVC% and comparing clinical and home spirometry, shows more scatter around the identity line, indicating more variability. Some data points significantly deviate from the line, suggesting home spirometry may be less consistent in measuring FEV1/FVC than FVC or FEV1 alone.

Home and clinical spirometry measurements showed strong correlations across all time points (Table 4). The correlation coefficients were consistently high for FVC, FEV_1_, and FEV_1_/FVC, as well as their respective z-scores, indicating a strong relationship between the two methods. All correlations were statistically significant (*p* < 0.0001). In Figure 2A,B, which illustrate FVC and FEV_1_ correlations, data points cluster near the identity line, reflecting a strong agreement between home and clinical spirometry. This suggests that home spirometry is a reliable alternative for tracking these parameters over time. However, in Figure 2C, which represents FEV_1_/FVC correlations, there is noticeably more scatter around the identity line, indicating greater variability between the two methods. Some data points significantly deviate from the line, suggesting that home spirometry may be less consistent in measuring FEV_1_/FVC compared to FVC or FEV_1_ alone.

### 3.5. Validity of Home Spirometry

FVC, FEV_1_, and FEV_1_/FVC measurements from clinical and home spirometry at weeks 4, 8, and 12 are presented in Table 5. The values obtained from home spirometry closely aligned with clinical spirometry results across all time points. The absolute mean differences between clinical and home spirometry measurements (clinical spirometry – home spirometry) were small. For FVC, the mean differences were −0.18 ± 0.22 mL at week 4 (*p* = 0.66), 0.14 ± 0.21 mL at week 8 (*p* = 0.72), and −0.05 ± 0.15 mL at week 12 (*p* = 0.08). Similarly, for FEV_1_, the absolute mean differences were 0.00, 0.02, and 0.00 at weeks 4, 8, and 12. For FEV_1_/FVC, the absolute mean differences were 0.58 ± 3.08% at week 4 (*p* = 0.31), 0.09 ± 3.12% at week 8 (*p* = 0.72), and 1.02 ± 2.46% at week 12 (*p* = 0.03). These findings indicate that home spirometry provided measurements comparable to clinical spirometry, with minor variations across different time points. Notably, the FEV_1_/FVC difference at week 12 reached statistical significance (*p* = 0.03), suggesting a slight discrepancy between clinical and home assessments in this parameter at that time point.

The z-score measurements for FVC, FEV_1_, and FEV_1_/FVC from clinical and home spirometry at weeks 4, 8, and 12 are presented in Table 6. Home spirometry demonstrated strong agreement with clinical spirometry for FVC z-scores, with minimal absolute mean differences across all time points. The differences were not statistically significant at weeks 4 and 8, but a significant difference was observed at week 12 (*p* = 0.021), see Table 6. The statistically significant difference observed in FVC z-score at week 12 may indicate slight variability in long-term monitoring, warranting further investigation. The mean differences between clinical and home spirometry FEV_1_ z-scores were minimal at all-time points. These differences were not statistically significant (*p* = 0.909, *p* = 0.772, *p* = 0.085, respectively). Home spirometry closely matched clinical spirometry FEV_1_/FVC z-scores, with values at weeks 4, 8, and 12. The absolute mean differences were small, and none were statistically significant (Table 6).

### 3.6. Improvement in Pulmonary Functions over Time

**Figure 3:**
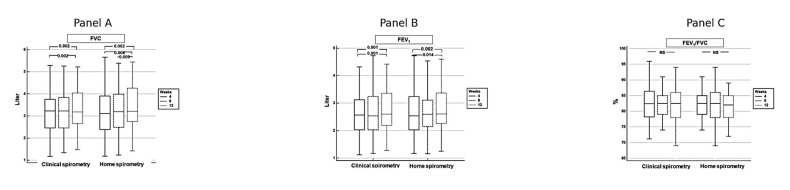
Improvement in pulmonary functions over time. Panel (**A**) (FVC): Both clinical and home spirometry showed statistically significant improvements in FVC between weeks 4, 8, and 12. The increase from week 4 to week 12 was significant (*p* = 0.002 for clinical spirometry, *p* = 0.002 for home spirometry), indicating improved lung capacity over time. Panel (**B**) (FEV_1_): A similar trend was observed for FEV_1_, with significant improvements from week 4 to week 12 in both clinical (*p* = 0.001) and home spirometry (*p* = 0.002), suggesting progressive recovery of airway function. Panel (**C**) (FEV_1_/FVC Ratio): No significant changes (NS) were observed in the FEV_1_/FVC ratio over time in either clinical or home spirometry, indicating consistent airflow dynamics despite improvements in absolute lung volumes.

Both home and clinical spirometry detected significant improvements in FVC and FEV_1_ over the 12-week follow-up period (Figure 3). From week 4 to week 12, FVC and FEV_1_ showed statistically significant increases in both measurement methods (*p* = 0.002 for FVC, *p* = 0.001–0.002 for FEV_1_, Table 2). These findings suggest a gradual recovery of lung function in post-COVID patients over time.

Both clinical and home spirometry showed statistically significant improvements in FVC between weeks 4, 8, and 12. The increase from week 4 to week 12 was significant (*p* = 0.002 for clinical spirometry, *p* = 0.002 for home spirometry), indicating improved lung capacity over time (Figure 3A). A similar trend was observed for FEV_1_, with significant improvements from week 4 to week 12 in both clinical (*p* = 0.001) and home spirometry (*p* = 0.002), suggesting progressive recovery of airway function. (Figure 3B). No significant changes were observed in the FEV_1_/FVC ratio over time in either clinical or home spirometry, indicating consistent airflow dynamics despite improvements in absolute lung volumes (Figure 3C). These results confirm progressive improvement in pulmonary function over 12 weeks post-COVID, with home and clinical spirometry providing comparable assessments.

### 3.7. Patient Satisfaction with Home vs. Clinical Spirometry

At week 12, participants completed a questionnaire assessing their experiences with home and clinical spirometry (Table 7). A total of 51.7% reported anxiety when performing home spirometry, compared to 31.0% in the clinic (*p* = 0.07). A total of 41.4% experienced coughing or fatigue after home spirometry, which was similar to clinical spirometry. No significant differences in mouthpiece and nose clip discomfort, shortness of breath, or dry mouth were reported between home- and clinic-based testing. Overall, home spirometry was well tolerated, with patient-reported experiences comparable to clinical spirometry.

## 4. Discussion

This study evaluated the clinical validity of an ultrasonic home-based spirometer for monitoring pulmonary function in post-COVID-19 pneumonia patients over 12 weeks. The findings indicate that home spirometry provides reliable and reproducible measurements comparable to those obtained through clinical spirometry, supporting its use as an effective tool for remote respiratory monitoring in this patient population.

Patient adherence to home spirometry was remarkably high throughout the study, with an average adherence rate of 98% over the 12 weeks. This strong compliance underscores the practicality and acceptability of home spirometry for patients recovering from COVID-19 pneumonia. The convenience of performing spirometry at home likely contributed to this adherence by reducing the need for frequent hospital visits, thereby alleviating the burden on both patients and healthcare systems.

In contrast, Ilic and colleagues reported lower compliance rates in interstitial lung disease patients, ranging from 40% to 98%, with an average of 69% over a 24-week follow-up period [23]. Similarly, Noth et al. observed a decline in adherence to home spirometry over time in idiopathic pulmonary fibrosis patients, though adherence remained above 75% throughout the 52-week INMARK trial [29]. A key factor in the high adherence observed in our study was the close follow-up implemented by our research team. Participants were instructed to perform home spirometry once weekly on a predetermined day, completing 3–8 maneuvers per session in the morning. Adherence was monitored in real-time by a research nurse via a cloud platform, with reminders sent to patients if measurements were missing. Additionally, the application provided real-time guidance, instructions, and feedback to ensure acceptable and repeatable maneuvers.

It remains to be seen whether this high level of compliance would persist over a longer follow-up period, as in Ilic’s study. Another contributing factor to adherence may have been the study population’s acceptance capacity for eHealth technology. All participants owned smartphones and willingly enrolled, demonstrating a readiness for telehealth applications. Notably, 47% of participants were university graduates, which may have further facilitated the adoption and effective use of the telehealth platform.

When the quality grades of the performed maneuvers were analyzed, we observed that clinical spirometry consistently achieved high-quality test sessions, with over 50% of tests graded as A across all visits. Home spirometry also demonstrated strong test quality, with a majority of sessions graded as A or B. However, slightly lower adherence to the highest-quality criteria (Grade A) was observed in some cases. The presence of a small number of Grade F tests in home spirometry suggests that some participants may have encountered challenges in performing maneuvers correctly, emphasizing the importance of continuous guidance and training. Overall, the quality of home spirometry was comparable to clinical spirometry, supporting its feasibility for remote pulmonary monitoring. However, studies on unsupervised home spirometry have reported varying results. A study on asthma and COPD patients in primary care found that only 59% of participants produced acceptable spirometry, defined as at least two repeatable measurements within 150 mL variability [30]. In contrast, Bell et al. reported that 93% of participants achieved Grade A or B spirometry without supervision, compared to 95% with supervision. Notably, Bell’s study population consisted of cystic fibrosis patients, who may have had prior experience with spirometry, potentially contributing to the high-quality results [24]. A meta-analysis of 28 studies that quantitatively compared unsupervised and supervised spirometry found that unsupervised testing yielded lower FEV_1_ and FVC values with substantial variability. The mean difference for FEV_1_ was −107 mL (LoA: −509 to 296; *p* < 0.001) and for FVC was −184 mL (LoA: −1028 to 660; *p* < 0.001) [20]. These findings highlight the critical role of remote supervision in ensuring spirometry quality in home-based settings. Importantly, our study presents a successful supervision methodology that effectively tracks patients during home spirometry, demonstrating a practical approach to maintaining measurement reliability.

The SpiroHome ultrasonic spirometer has been clinically validated for its accuracy and repeatability in measuring key pulmonary function parameters, including forced vital capacity (FVC) and forced expiratory volume in the first second (FEV_1_). In a study comparing the SpiroHome Clinic to the EasyOne Air spirometer, both devices met the accuracy requirements specified in relevant guidelines and standards [22]. The study found a strong correlation between the two devices, with a Pearson’s correlation coefficient of 0.99 for both FEV_1_ and FVC measurements. Bland–Altman plots demonstrated good agreement, with the majority of measurements falling within the 95% limits of agreement [22]. While specific data on the SensorMedics spirometers’ limits of agreement with home devices like SpiroHome are not readily available, it is important to note that acceptable limits of agreement between clinical and home spirometry devices for FVC and FEV_1_ are generally considered to be within ±150 mL or ±5% of the clinical measurement [25]. In our study, the Bland–Altman analysis demonstrated minimal bias between home and clinical spirometry measurements, showing that home spirometry provides similar measurements with clinical spirometry. This suggests that home spirometry does not systematically overestimate or underestimate lung function parameters compared to clinical spirometry. Furthermore, intraclass correlation coefficients (ICC) were very high, exceeding 99% for FVC, 96% for FEV_1_, and 92% for the FEV_1_/FVC ratio, confirming strong test–retest reliability. This suggests that home spirometry could be valuable for longitudinal monitoring, potentially reducing the need for frequent hospital visits. However, the limits of agreement varied, with FVC ranging from 450 mL to 420 mL and FEV_1_ from −360 mL to 360 mL (Table 2). These values exceed the commonly accepted 150 mL threshold, limiting the interchangeability of home and clinical spirometers. Similar findings have been reported in previous studies, which also observed high correlation coefficients and small mean differences but wide LoAs [20,24,31,32,33,34]. A meta-analysis by Anand et al. reported LoAs for FEV_1_ ranging from −509 mL to 296 mL and for FVC from −1028 mL to 660 mL. Based on these findings, the authors concluded that clinicians cannot confidently assume that home and clinical spirometry results are identical, with a 39% probability that the difference in FEV_1_ would exceed 200 mL when measured by clinical spirometry [20]. These findings underscore the potential utility of home spirometry for trend monitoring while highlighting its limitations in direct interchangeability with clinical devices due to the observed variability in agreement.

It is important to note that while most studies focus on FEV_1_ and FVC, research on FEV_1_/FVC in home spirometry remains limited [19,20,23,29,30,32,34]. In our study, we observed that the interclass and Pearson’s correlation coefficients were lower for FEV_1_/FVC and for the z-scores of FEV_1_/FVC compared to those for FEV_1_ and FVC (Table 2, Table 3 and Table 4, Figure 2). Unlike, FVC and FEV_1_, for the scatterplot of FEV1/FVC measurements between clinical and home spirometry, we observed more scatter around the identity line, indicating greater variability between the two methods. Gerbase et al. reported that systemic deviations varied between FEV_1_ and FVC, highlighting the need to consider potential biases in the FEV_1_/FVC ratio when evaluating agreement between spirometers in their study on handheld spirometer accuracy [35]. Therefore, we believe that future studies and meta-analyses should evaluate the validity and reliability of home spirometers in measuring FEV_1_/FVC, ensuring a more comprehensive understanding of their accuracy and clinical utility. Likewise, we found that ICCs and Pearson’s correlation coefficients were lower for z-scores compared to the corresponding absolute measurements of FVC, FEV_1_, and FEV_1_/FVC. To the best of our knowledge, our study is one of the first to evaluate z-scores in home spirometry. Z-scores remain underrepresented in home spirometry research, likely because most devices do not measure them, prioritizing ease of use and patient comprehension. However, as the new approach recommends z-score-based diagnosis for spirometry interpretation [36], future research should explore the integration of z-scores into home spirometry, enhancing diagnostic accuracy and improving clinical decision-making.

These results align with previous studies that have validated the use of home spirometry in various respiratory conditions. For instance, studies have shown that home spirometry correlates well with standard laboratory-based spirometry, offering high reliability and ease of use for patients with chronic respiratory diseases such as asthma, COPD, cystic fibrosis, and idiopathic pulmonary fibrosis [29,34,37]. Notably, home spirometry is highly accurate in patients with relatively normal lung function and offers advantages such as improved accessibility, reduced costs, and minimized infection risk compared to in-clinic spirometry [19,22,24]. However, data on its validity and clinical utility in post-COVID patients remain limited. Throughout the 12-week follow-up period in our study, the absolute mean differences between clinical and home spirometry measurements remained minimal. Home spirometry results closely matched those obtained from clinical spirometry, with only slight variations observed over time. However, at week 12, the FEV_1_/FVC difference reached statistical significance (*p* = 0.03), indicating a minor discrepancy between clinical and home-based assessments for this parameter. The challenges posed by the COVID-19 pandemic accelerated the adoption of eHealth technologies for remote healthcare delivery [17]. Our findings contribute to this growing body of research by demonstrating the feasibility and accuracy of home spirometry in monitoring lung function recovery among COVID-19 survivors.

In their multicenter, prospective 52-week trial, Noth et al. reported that while FVC measurements at individual time points from home and clinic spirometry were strongly correlated, the correlation between changes in FVC over time (i.e., trends or progression) was weak, primarily due to variability in home spirometry measurements [29]. In our study, we observed significant improvements in FVC and FEV_1_ over the 12-week follow-up period, as detected by both home and clinical spirometry. This indicates a gradual recovery of lung function in post-COVID-19 patients, highlighting the utility of home spirometry in tracking pulmonary recovery progress. The ability to monitor lung function remotely allows for timely interventions in cases where recovery may be delayed or complications arise.

In terms of patient satisfaction, home spirometry was well tolerated, with experiences comparable to clinical spirometry. While some patients reported anxiety when performing home spirometry, the overall discomfort and adverse effects were similar between home and clinical settings. This suggests that with proper training and support, patients can effectively perform spirometry at home without significant issues.

The findings of this study have important implications for the management of post-COVID-19 patients. The strong agreement between home and clinical spirometry measurements supports the integration of home spirometry into post-COVID-19 rehabilitation programs for routine lung function tracking. This approach can facilitate early detection of pulmonary function deterioration, reducing patient visits and allowing for prompt medical interventions and personalized rehabilitation strategies.

However, it is important to acknowledge the limitations of this study. The sample size was relatively small, and the study population was limited to patients with a specific severity of COVID-19 pneumonia. Future research should include larger and more diverse populations to validate these findings further. Additionally, while the study demonstrated high adherence to home spirometry, it did not assess the long-term sustainability of this adherence beyond the 12-week period. Further studies are needed to evaluate the long-term feasibility and effectiveness of home spirometry in monitoring pulmonary function in post-COVID-19 patients.

## 5. Conclusions

In conclusion, this study supports the use of home spirometry as a valid and reliable tool for monitoring lung function recovery in post-COVID-19 pneumonia patients. Home spirometry demonstrated strong agreement with clinical spirometry, particularly for FVC and FEV_1_ measurements, while FEV_1_/FVC ratios showed slightly more variability. Despite this, home spirometry remains a practical and convenient alternative for pulmonary function assessment, supporting its integration into long-term respiratory monitoring. The high patient adherence and reproducibility observed in this study highlight home spirometry’s potential role in enhancing patient monitoring, reducing healthcare burdens, and improving patient outcomes. However, given the large limits of agreement observed in FVC and FEV_1_ measurements, home spirometry should be positioned as a complementary tool for telemonitoring rather than a direct replacement for clinical spirometry. Further research is needed to determine the optimal integration of home spirometry in clinical practice, particularly in detecting clinical deteriorations and guiding personalized treatment strategies.

## Figures and Tables

**Table 1:** Characteristics of the study subjects.

	*n* = 30
Age, years, mean (SD)	48.64 ± 10.49
Male, *n* (%)	21 (70)
Height, m, mean (SD)	167.50 ± 10.41
Body mass index, kg/m^2^, mean (SD)	27.60 ± 8.16
FVC, L, mean (SD)	3.20 ± 0.99
FVC, predicted %, mean (SD)	83.93 ± 22.11
FVC, z-score, mean (SD)	−1.62 ± 1.86
FEV_1_, L, mean (SD)	2.60 ± 0.75
FEV_1_, predicted%, mean (SD)	82.79 ± 18.02
FEV_1_, z-score, mean (SD)	−1.46 ± 1.55
FEV_1_/FVC, %, mean (SD)	104.36 ± 7.49
FEV_1_/FVC, z-score, mean (SD)	0.35 ± 0.96

Data are expressed as mean ± standard deviation (SD) for continuous variables and as frequency (percentages) for categorical variables.

**Table 2:** Agreement between clinical and home spirometry: mean differences, limits of agreement, and intraclass correlation coefficients.

	The Mean Difference Between Clinic–Home Spirometry 95% CI	LoA Mean ± 2 SD	ICC 95% CI
FVC, best measurement, L, week 4	−0.02 −0.10–0.06	−0.45–0.42	0.989 0.976–0.995
FVC, best measurement, L, week 8	0.01 −0.06–0.09	−0.40–0.42	0.989 0.978–0.995
FVC, best measurement, L, week 12	−0.05 −0.11–0.01	−0.35–0.25	0.994 0.988–0.997
FEV_1,_ best measurement, L, week 4	0.00 −0.07–0.07	−0.36–0.36	0.987 0.972–0.994
FEV_1,_ best measurement, L, week 8	0.02 −0.03–0.07	−0.27–0.31	0.991 0.982–0.996
FEV_1,_ best measurement, L, week 12	−0.01 −0.05–0.03	−0.25–0.24	0.963 0.923–0.983
FEV_1_/FVC, best measurement, %,week 4	0.58 −0.58–1.72	−5.48–6.63	0.932 0.856–0.967
FEV_1_/FVC, best measurement, week 8	0.09 −1.07–1.26	−6.00–6.20	0.928 0.848–0.966
FEV_1_/FVC, best measurement, week 12	1.02 0.10–1.94	−3.80–5.84	0.941 0.876–0.972

This table presents the mean differences (95% confidence intervals) between clinical and home spirometry measurements for FVC, FEV_1_ and FEV_1_/FVC at weeks 4, 8, and 12. The limits of agreement (LoA) represent the range within which 95% of differences are expected to fall. Intraclass correlation coefficients (ICC) with 95% confidence intervals assess the reliability and agreement between the two spirometry methods.

**Table 3:** Agreement between clinical and home spirometry: mean differences, limits of agreement, and intraclass correlation coefficients for z-scores of spirometric data.

	The Mean Difference Between Clinic–Home Spirometry 95% CI	LoA Mean ± 2 SD	ICC 95% CI
z-FVC, week 4	−0.04 −0.32–0.23	−1.43–1.34	0.866 0.796–0.936
z-FVC, week 8	−0.04 −0.28–0.20	−1.25–1.16	0.90 0.85–0.96
z-FVC, week 12	−0.24 −0.45–−0.04	−1.28–0.79	0.914 0.87–0.96
z-FEV1, week 4	−0.015 −0.29–0.26	−1.38–1.35	0.81 0.71–0.91
z-FEV1, week 8	−0.033 −0.26–0.20	−1.2–1.13	0.861 0.790–0.933
z-FEV_1_, week 12	−0.18 −0.38–0.03	−6.32–−1.64	0.446 0.156–0.736
z-FEV1/FVC, %, week 4	0.24 −0.15–0.62	−1.71–2.19	0.472 0.195–0.748
z-FEV1/FVC, week 8	−0.02 −0.27 −0.27	−1.37–1.365	0.700 0.533–0.854
z-FEV1/FVC, week 12	0,11 −0.05–0.27	−0.68–0.9	0.831 0.741–0.921

This table presents the mean differences (95% confidence intervals) between clinical and home spirometry measurements for z-scores of FVC, FEV_1_ and FEV_1_/FVC at weeks 4, 8, and 12. The limits of agreement (LoA) represent the range within which 95% of differences are expected to fall. Intraclass correlation coefficients (ICC) with 95% confidence intervals assess the reliability and agreement between the two spirometry methods for normalized spirometric values.

**Table 4:** Correlations between spirometry variables measured at home and the clinic at weeks 4, 8, and 12.

	CS—FVC vs HS—FVC	CS—FVC-z vs HS—FVC- z	CS—FEV_1_ vs HS—FEV_1_	CS—FEV_1_-z vs HS—FEV_1_- z	CS—FEV_1_/FVC vs HS—FEV_1_/FVC	CS—FEV_1_/FVC-z vs HS—FEV_1_/FVC- z
Week 4	0.983 *	0.931 *	0.981 *	0.900 *	0.887 *	0.814 *
Week 8	0.981 *	0.948 *	0.985 *	0.928 *	0.895 *	0.833 *
Week 12	0.990 *	0.956 *	0.929 *	0.943 *	0.919 *	0.912 *

Pearson correlation coefficients denote positive strong correlations, * refers to *p* < 0.0001.

**Table 5:** Comparison of mean spirometric values between clinical and home spirometry at weeks 4, 8, and 12.

	Week 4	Week 8	Week 12
FVC, mL, mean ± SD			
Clinical spirometry	3.20 ± 0.99	3.27 ± 0.99	3.32 ± 0.99
Home spirometry	3.22 ± 1.09	3.25 ± 1.05	3.37 ± 1.05
Difference, absolute, mean ± SD	−0.18 ± 0.22	0.14 ± 0.21	−0.05 ± 0.15
*p*	0.66	0.72	0.08
FEV_1_, mL, mean ± SD			
Clinical spirometry	2.60 ± 0.75	2.65 ± 0.77	2.71 ± 0.74
Home spirometry	2.60 ± 0.85	2.62 ± 0.82	2.72 ± 0.82
Difference, absolute, mean ± SD	0.00 ± 0.18	0.02 ± 0.15	−0.00 ± 0.13
*p*	0.97	0.43	0.75
FEV_1_/FVC, %, mean ± SD			
Clinical spirometry	82.31 ± 5.50	81.72 ± 5.18	82.22± 4.48
Home spirometry	81.73 ± 6.64	81.63 ± 6.72	81.20 ± 5.83
Difference, absolute, mean ± SD	0.58 ± 3.08	0.09 ± 3.12	1.02 ± 2.46
*p*	0.31	0.72	0.03

This table presents the mean values (±standard deviation) for FVC, FEV_1_, and FEV_1_/FVC measured using clinical and home spirometry at weeks 4, 8, and 12. Absolute mean differences between the two methods and corresponding *p*-values are reported to assess statistical significance.

**Table 6:** Comparison of z-scores for spirometric parameters between clinical and home spirometry at weeks 4, 8, and 12.

	Week 4	Week 8	Week 12
FVC, z-score			
Clinical spirometry	−1.62 ± 1.86	−1.52 ± 1.81	−1.41 ± 1.76
Home spirometry	−1.58 ± 1.92	−1.48 ± 1.93	−1.17 ± 1.79
Difference, absolute, mean ± SD	−0.44 ± 0.71	−0.04 ± 0.61	−0.24 ± 0.53
*p*	0.743	0.723	0.021
FEV_1_, z-score			
Clinical spirometry	−1.46 ± 1.55	−1.39 ± 1.56	−1.25 ± 1.58
Home spirometry	−1.45 ± 1.57	−1.36 ± 1.58	−1.08 ± 1.46
Difference, absolute, mean ± SD	−0.01 ± 0.69	−0.33 ± 0.59	−0.17 ± 0.52
*p*	0.909	0.772	0.085
FEV_1_/FVC, z-score			
Clinical spirometry	0.35 ± 0.96	0.24 ± 0.89	0.26 ± 0.84
Home spirometry	0.12 ± 1.37	0.25 ± 1.24	0.14 ± 0.98
Difference, absolute, mean ± SD	0.24 ± 0.99	−0.00 ± 0.69	0.11 ± 0.40
*p*	0.214	0.986	0.167

This table presents the mean z-scores (±standard deviation) for FVC, FEV_1_, and FEV_1_/FVC measured using clinical and home spirometry at weeks 4, 8, and 12. Absolute mean differences between the two methods and corresponding *p*-values are reported to assess statistical significance.

**Table 7:** Patient satisfaction survey comparing home vs. clinical spirometry.

Questions	Home Spirometry	Clinical Spirometry	*p*
Experienced anxiety when performing spirometry due to uncertainty about executing the maneuver accurately	15 (51.7%)	9 (31.0%)	0.070
To keep blowing even though you do not feel anything is coming out	10 (34.5%)	11 (37.9%)	0.375
Coughing during the maneuver	12 (41.4%)	10 (34.5%)	0.500
Feeling tired after the test	12 (41.4%)	10 (34.5%)	0.500
Concerns about shortness of breath due to the test	6 (20.7%)	5 (17.2%)	1.000
Feeling dizzy	8 (27.6%)	5 (17.2%)	0.250
Nose clip uncomfortable	8 (27.6%)	9 (31.0%)	1.000
Mouthpiece uncomfortable	5 (17.2%)	7 (24.1%)	0.625
Not given enough information about why the test is performed	2 (6.9%)	3 (10.3%)	1.000
Dryness in mouth	8 (27.6%)	8 (27.6%)	1.000
Not given enough information about how to perform the test	1 (3.4%)	0 (0%)	1.000
Lack of support from healthcare professionals running the test	1 (3.4%)	0 (0%)	0.453
Feel embarrassed during the test (e.g., being shouted at to blow)	2 (6.9%)	1 (3.4%)	1.000

Data are presented as *n* (%). The McNemar test was used to compare home spirometry with clinical spirometry for patient feedback.

## Data Availability

The data presented in this study is available on request from the corresponding author. Due to the terms of the ethics committee approval, which did not include provisions for data sharing, the data cannot be made publicly available.

## Abbreviations

The following abbreviations are used in this manuscript:

FVC	forced vital capacity
FEV_1_	forced expiratory volume at 1st second
COVID-19	Coronavirus disease 2019
PFT	pulmonary function test
DLCO	carbon monoxide diffusing capacity
COPD	chronic obstructive pulmonary disease
ATS	American Thoracic Society
ERS	European Respiratory Society
PCR	polymerase chain reaction
GLI	Global Lung Initiative
SD	standard deviation
CI	confidence interval
IQR	interquartile range
HS	home spirometry
CS	clinical spirometry
LoA	limits of agreement
ICC	intraclass correlation coefficient
Z	z-score

## Appendix A

### Appendix A.1. Quality Grades of Spirometry Performed with Clinical and Home Spirometry

**Table:**

	Quality Grading of The Test Session						
		A	B	C	D	E	F
**Clinical spirometry, week 4, n = 29**	FVC	16	12			1	
	FEV_1_	19	9	1			
**Clinical spirometry, week 8, n = 39**	FVC	21	7				1
	FEV_1_	23	6				
**Clinical spirometry, week 12, n = 29**	FVC	24	4	1			
	FEV_1_	23	5	1			
**Home spirometry,** **week 4, n = 30**	FVC	21	7			2	
	FEV_1_	20	8	1		1	
**Home spirometry,** **week 8, n = 30**	FVC	19	8	1		2	
	FEV_1_	25	2		1	2	
**Home spirometry, week 12, n = 30**	FVC	23	6			1	
	FEV_1_	19	10			1	

Grading the quality of test sessions was performed according to ATS/ERS standards. Grades A, B, C, D, E, and F refer to ≥3 acceptable measurements within 0.150 L, 2 acceptable measurements within 0.150 L, ≥2 acceptable measurements within 0.200 L, ≥2 acceptable measurements within 0.250 L, ≥2 acceptable measurements >0.250 L, and 0 usable and 0 acceptable measurements, respectively.
